# Corticosteroids for improving patient-relevant outcomes in HELLP syndrome: a systematic review and meta-analysis

**DOI:** 10.1186/s12884-024-06665-y

**Published:** 2024-07-18

**Authors:** Asmaa F. Kasem, Hamdy B. Alqenawy, Marwa A. Elgendi, Radwa R. Ali, Rania HM Ahmed, Mohammad N. Sorour, Khadiga MH Hegab, Rania G. El-skaan, Rowyna H. El Helw, Mohamed S. Elsewefy, Maya M. Abdelrazek, Yasser M. Elrefaey, Mohamed YG Albahaie, Mohamed H. Salama, Ashraf F. Nabhan

**Affiliations:** Department of Obstetrics and Gynecology, Faculty of Medicine, Ain Shams University, Ramses Street, Cairo, 11591 Egypt

**Keywords:** HELLP syndrome, Corticosteroids, Maternal death, Perinatal death, Platelet transfusion, Pulmonary edema, Acute kidney injury, Renal dialysis, Liver failure

## Abstract

**Background:**

We conducted this updated systematic review to assess the effects of corticosteroids vs. placebo or no treatment for improving patient-relevant outcomes in hemolysis, elevated liver enzymes and low platelets (HELLP) syndrome.

**Methods:**

CENTRAL, MEDLINE/PubMed, Web of Science, and Scopus, from the date of inception of the databases to February 3, 2024 were searched. Reference lists of included studies and systematic reviews were thoroughly searched. We included RCTs that enrolled women with HELLP syndrome, whether antepartum or postpartum, to receive any corticosteroid versus placebo or no treatment. No language or publication date restrictions were made. We used a dual independent approach for screening titles and abstracts, full text screening, and data extraction. Risk of bias was assessed in the included studies using Cochrane’s RoB 2 tool. Pairwise meta-analyses were conducted, where two or more studies met methodological criteria for inclusion. GRADE approach was used to assess certainty of evidence for the pre-specified outcomes.

**Results:**

Fifteen trials (821 women) compared corticosteroids with placebo or no treatment. The effect of corticosteroids is uncertain for the primary outcome i.e., maternal death (risk ratio [RR] 0.77, 95% confidence interval [CI] 0.25 to 2.38, very low certainty evidence). Out of 6 studies reporting maternal death, 5 were judged overall to have “low risk” of bias. The effect of corticosteroids is also uncertain for other important outcomes including pulmonary edema (RR 0.70, 95% CI 0.23 to 2.09), dialysis (RR 3, 95% CI 0.13 to 70.78), liver morbidity (hematoma, rupture, and failure; RR 0.22, 95% CI 0.03 to 1.83), or perinatal death (0.64, 95% CI 0.21 to 1.97) because of very low certainty evidence. Low certainty evidence suggests that corticosteroids have little or no effect on the need for platelet transfusion (RR 0.98, 95% CI 0.60 to 1.60) and may result in a slight reduction in acute renal failure (RR 0.67, 95% CI 0.40 to 1.12). Subgroup and sensitivity analyses showed results that were similar to the primary synthesis.

**Conclusions:**

In women with HELLP syndrome, the effect of corticosteroids vs. placebo or no treatment is uncertain for patient-relevant outcomes including maternal death, maternal morbidity, and perinatal death. These uncertainties regarding this critical question should be addressed by adequately powered rigorous trials.

**Systematic review registration:**

Center for Open Science, osf.io/yzku5.

**Supplementary Information:**

The online version contains supplementary material available at 10.1186/s12884-024-06665-y.

## Background

The syndrome of hemolysis, elevated liver enzymes and low platelets (HELLP) has an incidence of 2.5 per 1000 singleton deliveries and it complicates 20% of women diagnosed with severe pre-eclampsia [1, 2].

The pathophysiology of HELLP syndrome that is usually diagnosed between 27 and 37 weeks, is not completely understood [3].

The diagnosis depends on laboratory findings of microangiopathic hemolysis, thrombocytopenia, and elevated liver enzymes. Different investigators reported different thresholds of hematologic and biochemical values for diagnosis of the syndrome or for determining the prognosis [4, 5].

The presence of HELLP syndrome is associated with significant maternal mortality and morbidity including acute renal and liver failure [1]. Approximately 70% of pregnancies complicated by HELLP syndrome require preterm delivery, thus increasing perinatal morbidity and mortality [5].

Observational studies suggested that steroid treatment in HELLP syndrome may improve disordered maternal hematological and biochemical features and perhaps perinatal mortality and morbidity. Hypothesized mechanisms include suppressing endothelial activation and reducing cytokine production, thereby alleviating inflammatory and anti-angiogenic responses linked to the syndrome’s pathophysiology. Clinical trials examined the effects of corticosteroids for the treatment of maternal HELLP syndrome. Various regimens have been reported using prednisolone, dexamethasone, or betamethasone [2, 5–7].

Current practice and clinical guidelines require an updated evidence synthesis because the latest available synthesis was published in 2010 [8], new studies have been published, and the clinical question remains relevant to decision makers.

We conducted this systematic review to update the synthesized evidence regarding the effects of corticosteroids versus placebo or no treatment for improving outcomes in women with HELLP syndrome.

## Methods

### Protocol and registration

This systematic review was conducted following the methodological standards of Cochrane Handbook [9]. We prospectively registered the protocol in Open Science Platform (osf.io/yzku5). The full text of the protocol is available in an open access registry and as an online as Supplementary File 1. We reported the review using the Preferred Reporting Items for Systematic reviews and Meta-Analyses (PRISMA) standards [10]. The full checklist is available as Supplementary File 2.

### Eligibility criteria

We included published randomized controlled trials that recruited women with HELLP syndrome, confirmed by objective testing. We included studies comparing corticosteroids versus placebo or no treatment. The primary outcome measure was maternal death. Other outcomes included acute pulmonary edema; acute renal failure; dialysis, liver morbidity (hematoma, ruptured liver, and failure), need for platelet transfusion, and perinatal death.

### Information sources

A comprehensive literature search was initially conducted on September 20, 2023, by two authors (AFN, HBA) who are information experts. We did not impose language or other restrictions on any of the searches. We searched bibliographic databases (Cochrane Central Register of Controlled Trials (CENTRAL), MEDLINE/PubMed) and citation indexes (Web of Science and Scopus). We included the terms (HELLP Syndrome) AND (corticosteroids or glucocorticoids or Dexamethasone or Betamethasone or Prednisolone). The search strategy was updated on February 3, 2024, and new studies were not identified. The detailed exact strategy adapted for each database is provided in Supplementary File 3 and is available as an open access registry document. We peer-reviewed the search strategy using PRESS checklist [11], and further tested it with a set of known relevant, ‘gold standard’ reports. We also searched clinical trial registries (ClinicalTrials.gov and the World Health Organization International Clinical Trials Registry Platform) to identify ongoing trials. We finally searched reference lists and explored the cited-by logs of identified studies and previously published reviews.

### Study selection

All reports identified in the databases were imported to Bibtex library using Jabref version 5. After removing duplicates, two authors independently screened all titles and abstracts for eligibility. We retrieved and assessed the full text of all reports that potentially met our eligibility criteria during screening. Two authors (AFK, HBA, MAE, RHA) independently assessed each full-text article. Disagreements regarding trial eligibility were resolved by consensus and finally resolved by a third author (AFN).

### Data collection process

For eligible studies, we extracted the data in duplicates using an offline electronic form. We resolved discrepancies through discussion. Extracted data were transcribed to a spreadsheet and checked for accuracy. We contacted authors of the original reports, if needed, to provide details regarding unclear or missing data.

### Data items

Extracted data included study design, sample size, description of included participants, description of the intervention, outcomes, trial registration, and funding sources, and country.

### Study risk of bias assessment

Two authors (AFK, HBA, MAE, RHA) independently used the Risk of Bias 2 (RoB 2) tool to assess the risk of bias of study results contributing information to each of the outcomes specified for inclusion in the Summary of Findings table.

We assessed the following risk of bias domains as outlined in Cochrane Handbook for Systematic Reviews of Interventions: (1) risk of bias arising from the randomization process; (2) risk of bias due to deviations from the intended interventions (effect of assignment to intervention); (3) risk of bias due to missing outcome data; (4) risk of bias in measurement of the outcome; and (5) risk of bias in selection of the reported result. Each domain was judged as being at “low risk of bias”, “some concerns”, or “high risk of bias”. Trials with “low risk of bias” in all domains were classified as being at overall “low risk of bias”. RCTs with one domain judged to be at “some concerns”, but no domain judged to be at “high risk of bias”, were classified as being at overall “some concerns” of risk of bias. RCTs were classified as being at overall “high risk of bias” if at least one domain was judged as being at “high risk of bias”. However, if a trial was judged to be at “some concerns” due to risk of bias for multiple domains, it was judged as being at overall “high risk of bias” if the assessors judged that the multiple concerns amounted to a serious risk of bias. In case of discrepancies among their judgments and inability to reach consensus, we consulted the senior author (AFN) to reach a final decision.

We did not use any trial trustworthiness checklist.

### Effect measures

For dichotomous data, we presented results as summary risk ratio (RR) with 95% confidence intervals (CI). None of the outcomes of interest were meta-analyzed as a continuous variable. The unit of analysis was the individual participant. We used a complete-case approach for analysis. Data related to participants reported as not compliant was analyzed on an intention-to-treat basis.

### Synthesis methods

Fixed-effect meta-analysis was performed to combine data of trials that are judged to be sufficiently similar in terms of intervention, populations, and methods. We planned to investigate substantial statistical heterogeneity, defined as I² statistic ≥ 50% or *P* < 0.1.

We performed the planned subgroup analysis by gestational age at enrollment (ante- vs. postpartum) and by type of corticosteroids. We assessed subgroup differences by interaction tests. Results of the subgroup analyses were reported by mentioning the Chi² statistic and P value, and the interaction test I² value.

Sensitivity analysis was performed to explore robustness of pooled estimate using outcome data from trials with a low risk of bias.

Synthesis was performed using RStudio 2023.06.1 Build 524 (MacOS, Apple Silicon version), R 4.3.1 (2023-06-16) [12] and R package *meta* version 6.5 [13].

### Reporting bias assessment

We explored whether the study was included in a trial registry and whether a protocol was available. We planned to examine funnel plots to assess the potential for publication bias if we found 10 or more studies reporting on a particular outcome.

### Certainty assessment

We used the Grading of Recommendations, Assessment, Development and Evaluation (GRADE) approach to create the Summary of Findings Table [14] Briefly, GRADE uses study limitations, consistency of effect, imprecision, indirectness, and publication bias to assess the certainty of evidence for each outcome. A summary of the intervention effect and a measure of certainty was produced using the GRADE Profiler Guideline Development Tool (GRADEpro GDT) software [15] for the prespecified important outcomes: maternal death, pulmonary edema, renal failure, dialysis, liver morbidity, need for platelet transfusion, and perinatal death. One author (AFN) conducted GRADE assessments and the decisions on downgrading. This was discussed for final approval by all authors.

### Public involvement

We invited a consumer peer-review and feedback on the final draft prior to submission.

## Results

### Study selection

**Fig. 1:**
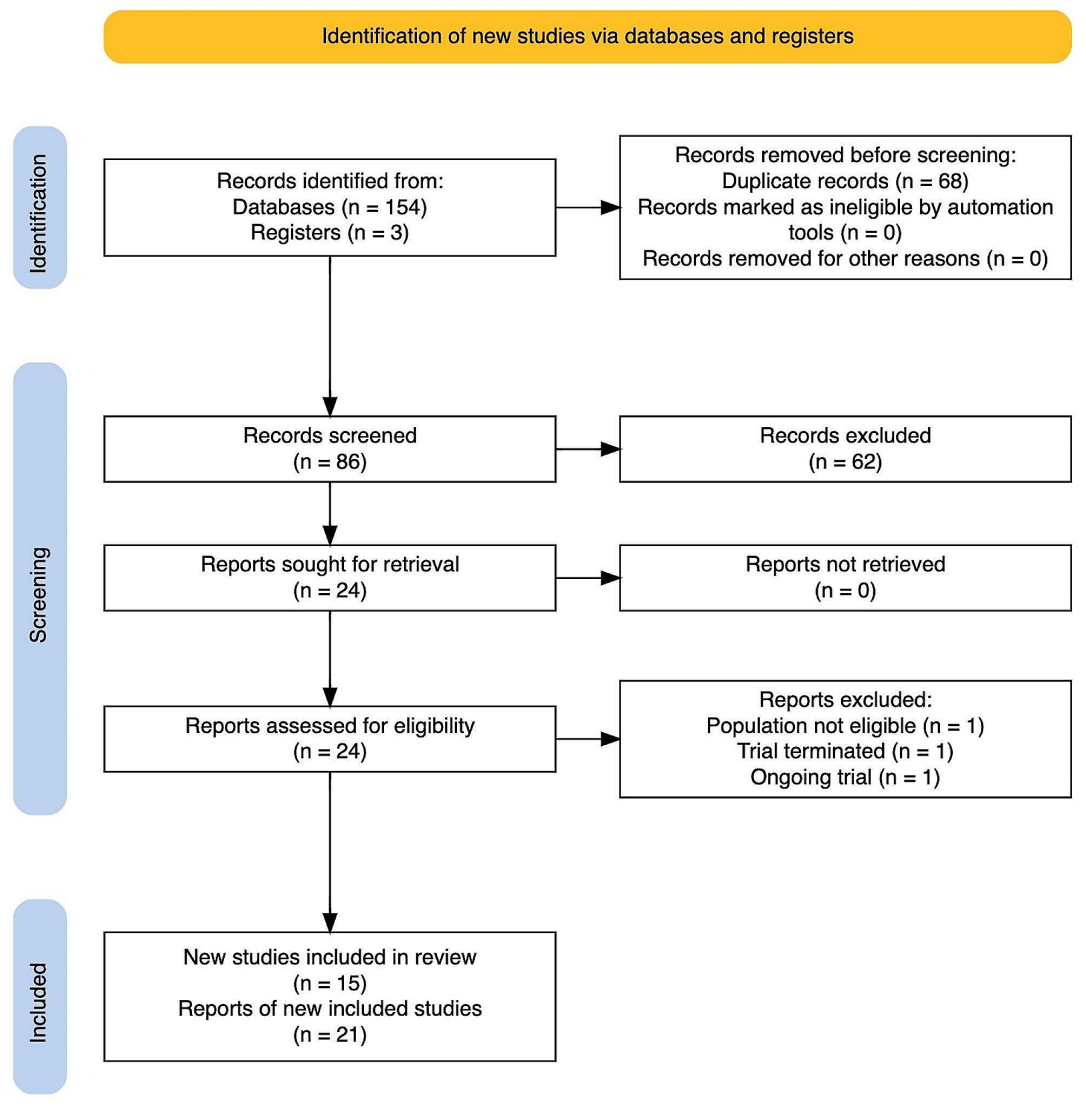
PRISMA flow diagram. PRISMA, Preferred Reporting Items for Systematic reviews and Meta-Analyses

Bibliographic database search identified 154 records. After removing duplicates, 86 titles and abstracts were screened. Twenty-four titles required further assessment. One was an ongoing trial, and the full-text reports of 23 published reports were assessed using the predefined eligibility criteria. We excluded two reports identified in our search. One study did not meet our inclusion criteria for participants as it enrolled women with low platelets not HELLP syndrome. The other study was terminated because of the inability to recruit the required sample. Reasons for exclusion of those three reports are detailed in Supplementary File 5. Fifteen studies (21 reports published between 1994 and 2019) were found eligible (Fig. 1).

### Study characteristics

We summarized the characteristics of the included studies in Table 1.

The 15 included trials recruited 821 women with HELLP syndrome. Criteria for recruitment in six trials [16–21] were a diagnosis of HELLP class 1 or 2 on the Mississippi HELLP classification system. One trial exclusively recruited women with class 1 [22]. One trial recruited women with class 2 and 3 [23]. Three studies [24–26] recruited women with HELLP classes 1, 2, and 3. One study [27] included women with partial HELLP (1 or more parameters abnormal) (61/105 [58.1%]) and complete HELLP (all parameters abnormal) (44/105 [41.9%]); class 1 and 2 combined subset accounted for 85.7% of participants with complete HELLP. Three studies [28–30] did not report explicitly on the class of HELLP syndrome.

Eleven trials administered dexamethasone vs. placebo or no treatment, [17–19, 21–23, 26–30] two trials administered betamethasone [24, 25], and one trial administered prednisolone [20]. One multiple-arms trial compared dexamethasone vs. betamethasone vs. no treatment [16].

Corticosteroids administration commenced after delivery in eight trials [16, 17, 19, 21, 26–28, 30], before delivery in five trials [20, 23–25, 29], and in two trials [18, 22] treatment commenced according to timing of recruitment whether before or after delivery. All women received the standard of care for management of severe preeclampsia, including magnesium sulfate and anti-hypertensive medications. Some studies [16, 18, 20, 22, 23, 25, 27, 30] clearly reported including women who received antenatal corticosteroids for fetal lung maturation apart from the study-specific steroid doses (Supplementary File 5).

**Table 1:** Characteristics of included studies

Study ID (Country)	Treatment commenced	Inclusion criterion according to GA or postpartum days	Sample size	Corticosteroids	HELLP syndrome class	Corticosteroids regimen
Bouchnak 2005 [28] (Tunisia)	Postpartum	Immediately after delivery	20	Dexamethasone	Not clearly defined	Postpartum women in the experimental group received dexamethasone, 12 mg every 12 h for 2 doses, started immediately following delivery.
Fonseca 2005 [18] (Colombia)	Antepartum and Postpartum	> 20 weeks, first 3 days postpartum	132	Dexamethasone	Class 1 (38%) and 2 (62%)	Pregnant women in the experimental group received 10 mg doses of dexamethasone intravenously every 12 h until delivery and 3 additional doses after delivery. Puerperal women received 3 10-mg doses after delivery.
Fonseca 2019 [22] (Colombia)	Antepartum and Postpartum	> 20 weeks, first 3 days postpartum	87 (out of prespecified 144 calculated sample)	Dexamethasone	Class 1	Pregnant women in the experimental group received 10 mg doses of dexamethasone, intravenously, every 12 h until delivery; and 3 additional doses after delivery. Postpartum women received three 10 mg doses after delivery.
Kadanali 1997 [29] (Turkey)	Antepartum	27–37 weeks	26	Dexamethasone	Not clearly defined	Pregnant women in the experimental group received a total of four doses of intravenous dexamethasone over 36 h separated by 12-hour intervals. The first two doses were 10 mg each and the second two doses were 5 mg each.
Katz 2008 [27] (Brazil)	Postpartum	Days not specified	105	Dexamethasone	Partial HELLP and complete HELLP. Class 1 and 2 combined subsets of complete HELLP is 85.7%	Postpartum women in the experimental group received 10 mg doses of dexamethasone every 12 h for 4 days. Extra doses may have been administered to an undisclosed number of women with deteriorated status.
Magann 1994 [23] (USA)	Antepartum	24–37 weeks	25	Dexamethasone	Class 2 and 3	Pregnant women in the experimental group received 10-mg doses of dexamethasone intravenously every 12 h until delivery.
Magann 1994 [19] (USA)	Postpartum	Immediate postpartum	40	Dexamethasone	Class 1 and 2	Postpartum women in the experimental group received a total of four doses of dexamethasone separated by 12-hour intervals starting immediately after delivery and throughout the following 36 h. The first two doses were 10-mg each and the second two doses were 5-mg each.
Mould 2006 [30] (South Africa)	Postpartum	Not specified	37	Dexamethasone	Not clearly defined	Postpartum women in the experimental group received 10 mg dexamethasone every 12 h until platelets recovered (> 100,000 cells/mm3).
Ozer 2009 [25] (Turkey)	Antepartum	> 20 weeks	60	Betamethasone	Class 1, 2 and 3	Pregnant women in the Intervention group received 12 mg betamethasone IM every 12 h until symptoms and signs in remission
van Runnard 2006 [20] (Netherlands)	Antepartum	< 30 weeks	32	Prednisolone	Class 1 and 2	Pregnant women in the interventions group received prednisolone IV, 50 mg over 12 h in 100 ml of sodium chloride, for 2 days after delivery or for up to 14 days in antenatal period, then tapering off (4-day oral tapering protocol of 50, 20, 10 and 5 mg of medication). If women delivered during the tapering period, a stress dose was given during and after delivery every 12 h for 48 h)
Vigil-De Gracia 1997 [26] (Mexico)	Postpartum	Immediate postpartum	34	Dexamethasone	Classes 1, 2, and 3	Postpartum women in the intervention group received 10 mg IV dexamethasone, repeated at 12 and 24 h (total 30 mg)
Yalcin 1998 [21] (Turkey)	Postpartum	Immediate postpartum	30	Dexamethasone	Class 1 and 2	Postpartum women in the intervention group received 10 mg dexamethasone IV, then 10 mg at 12 h, and 5 mg at 24 and 36 h (total dose over 36 h = 30 mg)
Du Plessis 2010 [17] (South Africa)	Postpartum	Not specified	68	Dexamethasone	Class 1 and 2	Postpartum women in the intervention group received dexamethasone 24 mg on day 1, 16 mg on day2 and 12 mg on day 3, intravenously
Borekci 2008 [16] (Turkey)	Postpartum	Not specified	60	Dexamethasone vs. Betamethasone vs. placebo	Class 1 and 2	The first group was given 10 mg dexamethasone intravenously three times with a 12-hour interval for a total dose of 30 mg. The second group was given 12 mg betamethasone intramuscularly twice with a 24-hour interval. It was administered at a total dose of 24 mg.
Caliskan 2010 [24] (Turkey)	Antepartum and Postpartum	27–37 weeks	65	Betamethasone	Class 1, 2 and 3	Pregnant women in the Intervention group received 24 mg betamethasone intramuscularly before cesarean delivery and was repeated 24 h later.

### Risk of bias in studies

We assessed the risk of bias for the included RCTs contributing results to our outcomes using the RoB 2 tool. The overall risk of bias for all study results per outcome are available in Supplementary File 4.

**Fig. 2:**
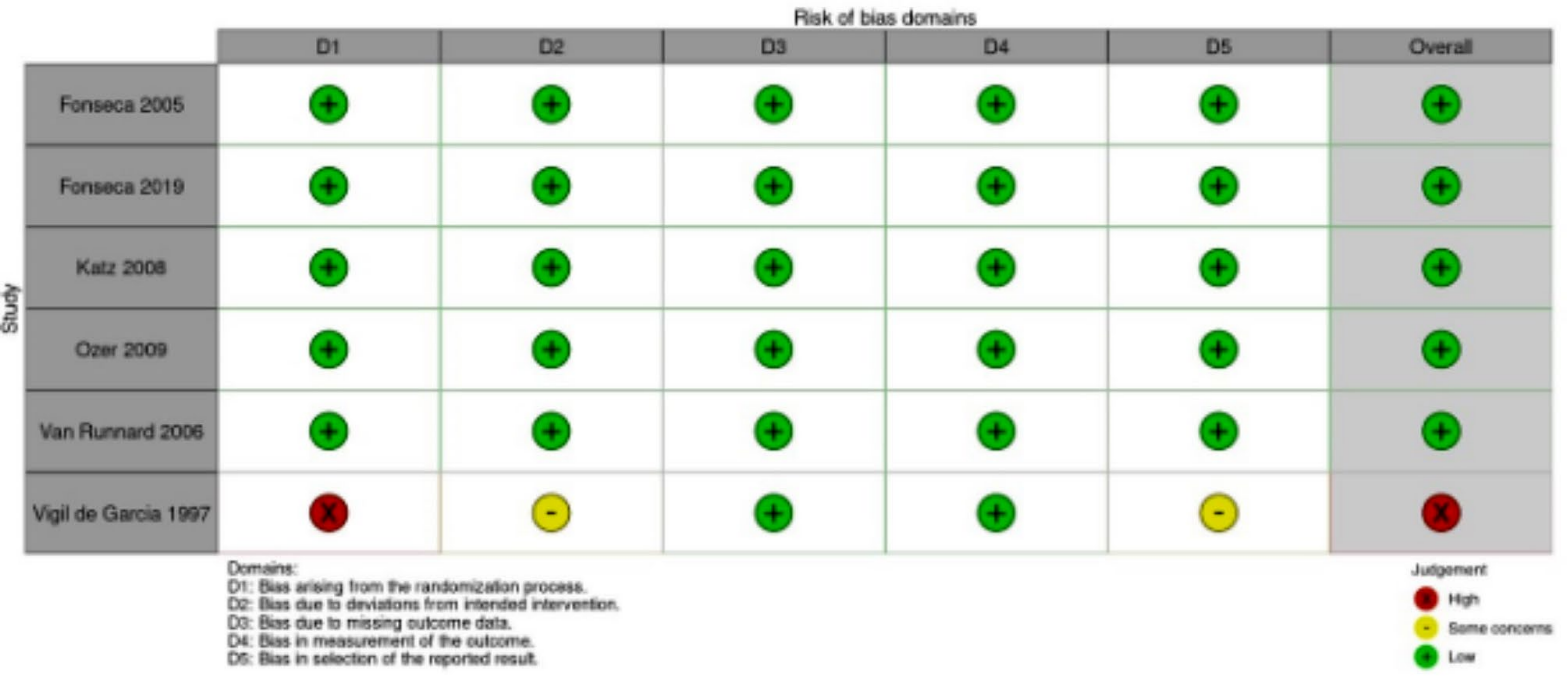
Risk-of-bias summary for maternal death: Corticosteroids vs. placebo or no treatment

The results of risk-of-bias assessment for the primary outcome, maternal death, are depicted in Fig. 2. Overall, 5 out of 6 studies were judged overall to be at “low risk” of bias. One study [26] was judged to be at “high risk” of bias. The main reasons for having “high risk” in domain 1 were lack of description in the randomization process with baseline differences in platelet count between intervention groups that suggest a problem with the randomization process. There were “some concerns” in two other domains. First, people delivering the interventions were probably aware of participants’ assigned intervention during the trial. Second, there were no information whether the data that produced this result were analyzed in accordance with a pre-specified analysis plan that was finalized before unblinded outcome data were available for analysis.

### Synthesis of results

#### Maternal death

**Fig. 3:**
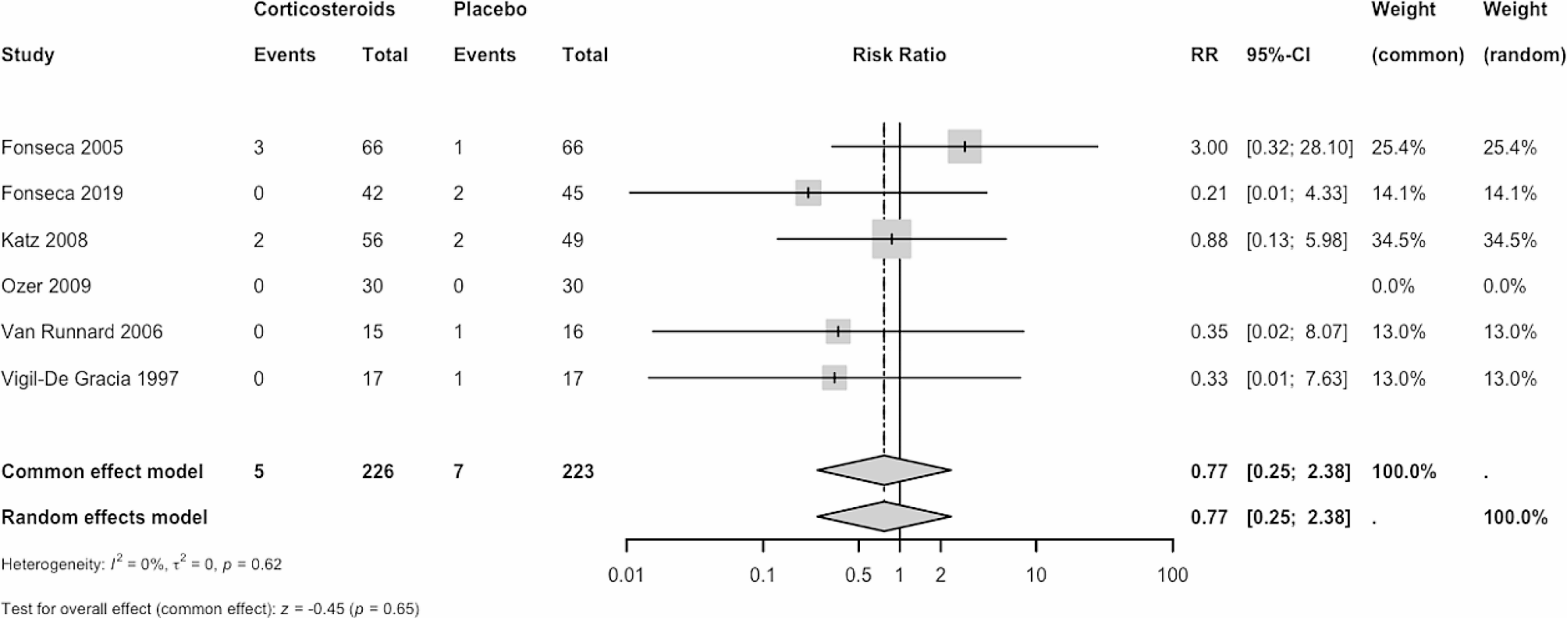
Forest plot for maternal death: Corticosteroids vs. placebo or no treatment

Six trials (449 women) reported maternal death. The risk ratio (RR) was 0.77 (95% confidence intervals (CI) 0.25 to 2.38 under both the fixed-effect (FE) and random-effects (RE) models, Fig. 3). The effect of any corticosteroid vs. placebo or no treatment is uncertain. We downgraded the certainty of the evidence to very low due to extremely serious imprecision (Table 2).

The subgroup analysis did not show significant differences among groups whether by the timing of corticosteroid administration (test for subgroup differences *P* = 0.79) or by the type of corticosteroid (test for subgroup differences *P* = 0.60) (Supplementary File 4).

Sensitivity analysis to explore robustness of pooled estimate for maternal death, using outcome data from trials with a low risk of bias showed results similar to primary analysis with a RR 0.87 (95% CI 0.26 to 2.92) or by including studies with zero events with RR 0.79 (95% CI 0.27 to 2.32) (Supplementary File 4).

#### Acute pulmonary edema

**Fig. 4:**
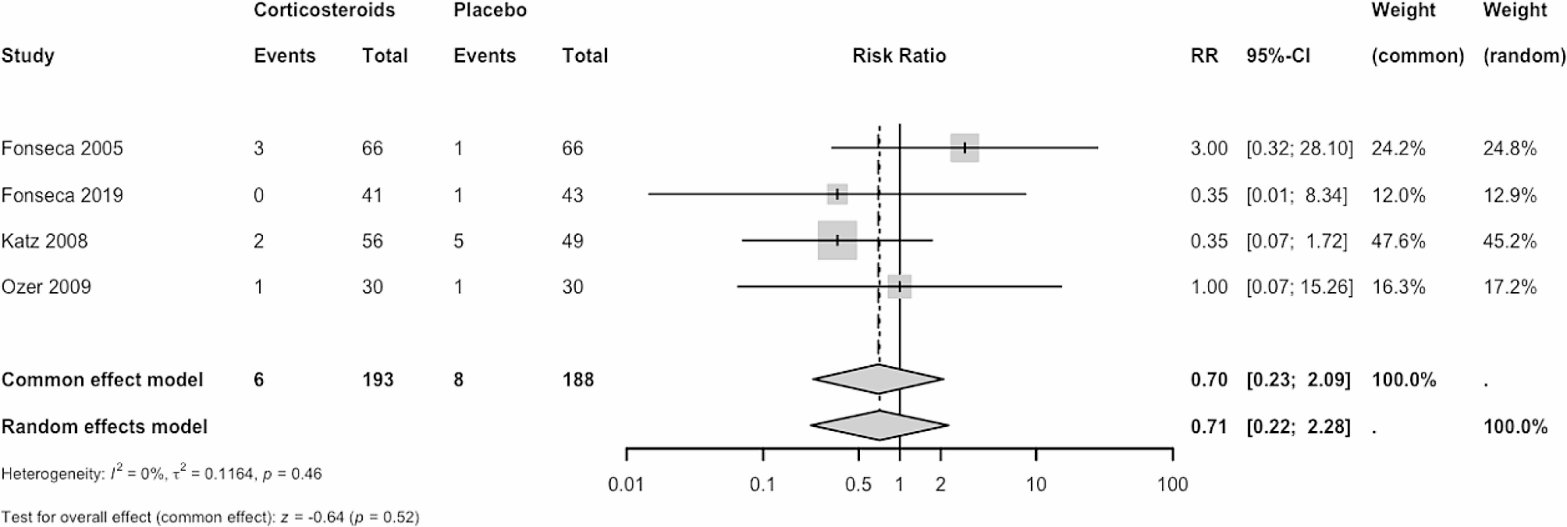
Acute pulmonary edema: Corticosteroids vs. placebo or no treatment

The effect of any corticosteroid vs. placebo or no treatment is very uncertain. Four trials (381 women) reported pulmonary edema. The RR was 0.70 (95% CI 0.23 to 2.09, FE; RR 0.71, 95% CI 0.22 to 2.28, RE), Fig. 4. We downgraded the certainty of the evidence to very low due to extremely serious imprecision.

#### Acute renal failure

**Fig. 5:**
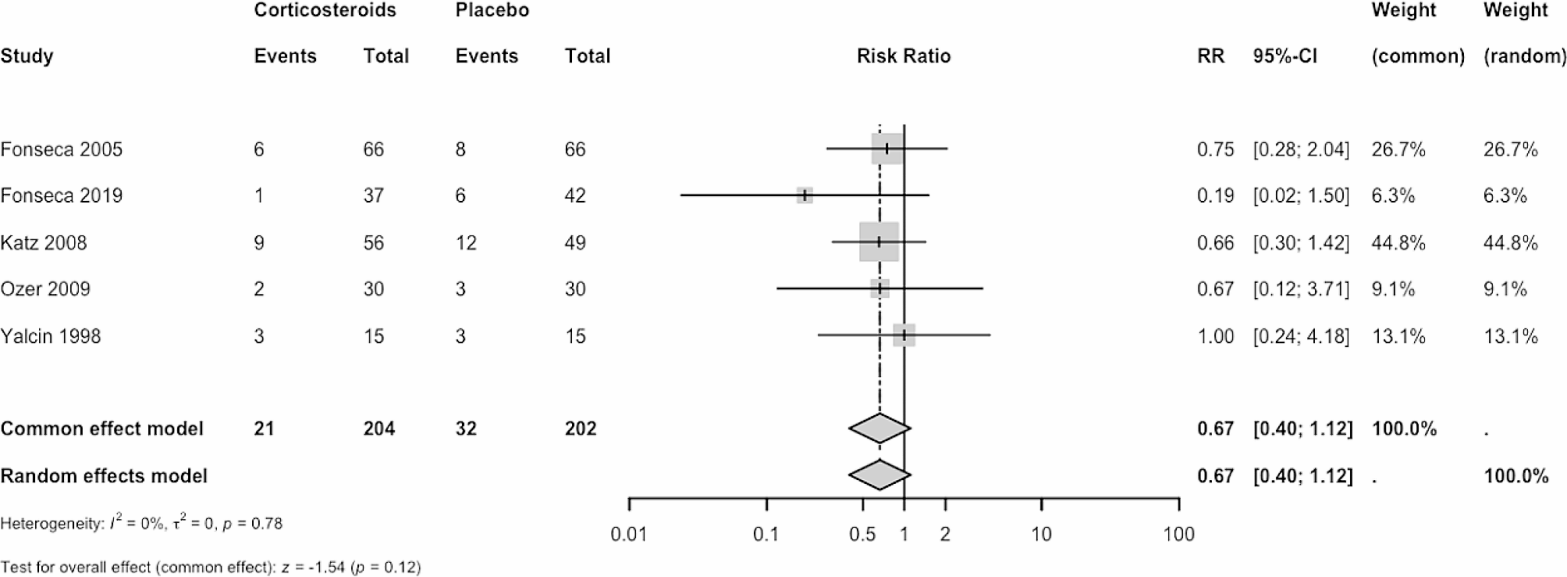
Acute renal failure: Corticosteroids vs. placebo or no treatment

Five trials (406 women) reported acute renal failure. Corticosteroids may result in a slight reduction in acute renal failure. The RR was 0.67 (95% CI 0.40 to 1.12 under both FE and RE models), Fig. 5. The certainty of evidence was low due to very serious imprecision.

#### Dialysis

The evidence is very uncertain about the effect of corticosteroids on dialysis. The need for dialysis was reported in one study (60 women). The RR was 3 (95% CI 0.13 to 70.78). The certainty of evidence was downgraded to very low due to extremely serious imprecision.

#### Liver morbidity (hematoma, rupture, or failure)

The evidence is very uncertain about the effect of corticosteroids on liver morbidity vs. placebo or no treatment. Based on data from two studies (91 women), the RR was 0.22 (95% CI 0.03 to 1.83 under both FE and RE models). We downgraded the certainty of evidence to very low due to extremely serious imprecision.

#### Platelet transfusion

Based on data from 219 women in two studies, corticosteroids have little or no difference in the need for platelet transfusion (RR 0.98; 95% CI 0.60 to 1.60 under both FE and RE models). We downgraded the certainty of evidence to low due to very serious imprecision.

#### Perinatal death

The evidence is very uncertain about the effect of corticosteroids on perinatal death. Based on data from two studies (58 women), the RR was 0.64 (95% CI 0.21 to 1.97 under both FE and RE models). The certainty of evidence was very low due to extremely serious imprecision.

### Risk of reporting biases in syntheses

The possibility of reporting bias could not be excluded, as not all trials reported all relevant outcomes. The planned funnel plots were not created because we did not include 10 or more studies reporting on any of the outcomes.

### Certainty of evidence

The effect of corticosteroids, compared with placebo or no treatment, is uncertain for maternal death, acute pulmonary edema, dialysis, and perinatal death. We downgraded the certainty of the evidence three levels to very low due to extremely serious imprecision. The 95% CI is very wide and includes both large benefit and large harm. The two boundaries of CI suggest very different inferences.

Corticosteroids, compared with placebo or no treatment, may result in a slight reduction in acute renal failure. We downgraded the certainty of the evidence two levels to low due to very serious imprecision. The 95% CI overlaps no effect and includes large benefit.

Corticosteroids, compared with placebo or no treatment, have little or no difference in the need for platelet transfusion. We downgraded the certainty of evidence two levels to low due to very serious imprecision. The pooled estimate of the risk ratio suggests no difference and the CI includes appreciable benefit and harm.

A Summary of Findings table presents the same information as the text above, with footnotes explaining judgments, Table 2.

**Table 2:** Summary of findings: corticosteroids compared to placebo for women with HELLP syndrome

Outcomes	Number of participants (studies) Follow-up	Certainty of the evidence (GRADE)	Relative effect (95% CI)	Anticipated absolute effects	
				Risk with placebo	Risk difference with Corticosteroid
Maternal death	449 (6 RCTs)	⨁◯◯◯ Very low^a^	**RR 0.77** (0.25 to 2.38)	31 per 1,000	**7 fewer per 1,000** (24 fewer to 43 more)
Acute Pulmonary edema	381 (4 RCTs)	⨁◯◯◯ Very low^a^	**RR 0.70** (0.23 to 2.09)	43 per 1,000	**13 fewer per 1,000** (33 fewer to 47 more)
Acute renal failure	406 (5 RCTs)	⨁⨁◯◯ Low^b^	**RR 0.67** (0.40 to 1.12)	144 per 1,000	**48 fewer per 1,000** (87 fewer to 17 more)
Dialysis	60 (1 RCT)	⨁◯◯◯ Very low^a^	**RR 3.00** (0.13 to 70.78)	0 per 1,000	**0 fewer per 1,000** (0 fewer to 0 fewer)
Liver morbidity	91 (2 RCTs)	⨁◯◯◯ Very low^a^	**RR 0.22** (0.03 to 1.83)	87 per 1,000	**68 fewer per 1,000** (85 fewer to 72 more)
Platelet transfusion	219 (2 RCTs)	⨁⨁◯◯ Low^c^	**RR 0.98** (0.60 to 1.60)	225 per 1,000	**4 fewer per 1,000** (89 fewer to 135 more)
Perinatal death	58 (2 RCTs)	⨁◯◯◯ Very low^a^	**RR 0.64** (0.21 to 1.97)	233 per 1,000	**85 fewer per 1,000** (185 fewer to 226 more)

The risk in the intervention group (and its 95% confidence interval) is based on the assumed risk in the comparison group and the relative effect of the intervention (and its 95% CI)

CI: confidence interval; RR: risk ratio; HELLP: Hemolysis, Elevated Liver enzymes, Low Platelets

### GRADE working group grades of evidence

- High certainty: we are very confident that the true effect lies close to that of the estimate of the effect.
- Moderate certainty: we are moderately confident in the effect estimate: the true effect is likely to be close to the estimate of the effect, but there is a possibility that it is substantially different.
- Low certainty: our confidence in the effect estimate is limited: the true effect may be substantially different from the estimate of the effect.
- Very low certainty: we have very little confidence in the effect estimate: the true effect is likely to be substantially different from the estimate of effect.

### Explanations

- CI is very wide and includes both large benefit and large harm. The two boundaries of CI suggest very different inferences. We rated down three levels for imprecision.
- CI overlaps no effect and includes both large benefit and small harm. We rated down two levels for imprecision.
- The pooled estimate of the risk ratio suggests no difference and the CI includes appreciable benefit and harm.

## Discussion

We conducted this systematic review and meta-analysis to assess the effects of corticosteroids for improving outcomes in women with HELLP syndrome. This updated evidence synthesis is mandatory for the development of the Egyptian National Guideline for the management of severe preeclampsia, commissioned by the Egyptian Health Council. In addition, new trials have been published in the decade after the previous Cochrane Review [8] in 2010. The clinical question, which is crucial for decision-making, remains not answered with high-certainty evidence.

### Summary of the evidence

There was no clear evidence of a treatment effect of corticosteroids on substantive clinical outcomes. The effect of corticosteroids, compared with placebo or no treatment, is uncertain for maternal death, acute pulmonary edema, dialysis, and perinatal death. Corticosteroids, compared with placebo or no treatment, have little or no difference in the need for platelet transfusion but may result in a slight reduction in acute renal failure.

The results of this up-to-date review are consistent with the findings reported previously [8] that there was insufficient evidence to support the administration of corticosteroids to women with HELLP syndrome.

In this review, we only included randomized trials for the meta-analysis. To ensure reliability of the results, observational studies were excluded due to their higher risk of bias when evaluating the effects of interventions. Published reviews [31] that included observational studies have not adequately addressed potential confounders and the likelihood of increased heterogeneity resulting from residual confounding and from other biases that vary across studies.

Our strategy aimed to study the effectiveness of corticosteroids in HELLP syndrome in improving critical maternal and perinatal outcomes rather than surrogate outcomes. Some interventions affecting these markers might have no or even harmful effects on clinical outcomes, while others with no effect on markers might still improve outcomes [32, 33]. As such, favorable effects on platelet count and liver enzymes, though appealing, may not reflect actual clinical benefits. The previous Cochrane Review [8] reported corticosteroid use for improving platelet count if raising the count was considered clinically worthwhile. The conclusion again refers to patient benefit even when considering a surrogate outcome. Furthermore, surrogate end points are potentially misleading and should be avoided, or at least interpreted with caution, as decision makers are required to extrapolate the findings to estimate true patient benefits, resulting in uncertainty. Published synthesized evidence [31] that included surrogate outcomes without downgrading the certainty of evidence for indirectness would provide misleading implications for practice. In the presence of patient-relevant outcomes, the use of surrogate outcomes in a synthesis of evidence to inform practice cannot be justified [34].

We focused on studies that compared corticosteroids to placebo or standard care. Various types of corticosteroids differ in their relative potency and duration of action. It would, therefore, be counter-intuitive, and not clinically useful, to compare one corticosteroid to another when evidence fails to show a difference between any corticosteroid vs. placebo or no treatment. Investigators [35] raised serious concerns regarding the credibility of the subgroup analysis results of the Cochrane Review [8] and the application of these subgroup results into clinical practice.

A recently published review [36] had serious concerns that stakeholders must be aware of. The review included a trial that enrolled women without HELLP syndrome, [37] while it did not include nine eligible published trials [16, 17, 19, 21, 24, 26, 28–30]. The meta-analyses suffered from incorrect data entry both in the number of events and in the total number of participants in several outcomes leading to incorrect estimates of the effects that have serious implications when interpreting the results [36]. Further, the review indicated that the revised Cochrane Risk of Bias tool (RoB 2) was used. RoB 2 is a “results-based” tool because it is used to assess bias for a specific result reported in an individual study. The original risk of bias tool, and most other tools, assess bias across all outcomes and results for an entire study. However, the review incorrectly reported the risk of bias for each entire trial while the RoB table headings and the citation refer to RoB-2. The review did not report any subgroup or sensitivity analyses. The review did not rate the certainty of evidence using any approach such as GRADE [36].

In summary, our methodology minimized bias through strict inclusion of randomized controlled trials, established the class effect first before agent comparisons, and emphasized outcomes of greatest clinical relevance. This approach provided the most robust and applicable evidence for clinical decision making.

The results of our up-to-date synthesis of available evidence provide a rigorous evidence base for trustworthy clinical practice guidelines for the management of HELLP syndrome [38–44].

### Limitations

A limitation of the evidence was the small number of eligible studies, and the restricted number of outcomes reported in the included trials. The small sample sizes in these trials resulted in imprecision, negatively impacting the certainty of evidence. There was also the possible confounding effect of antenatal corticosteroid administration for fetal lung maturation. In primary studies, the pragmatic inclusion criteria increased generalizability at the expense of precision. This was handled by prespecified subgroup and sensitivity analyses. Most included trials reported surrogate laboratory results. The possibility of reporting bias could not be excluded, given that not all trials reported all relevant outcomes. In the case of HELLP syndrome, patient-relevant outcomes do not require exceptional training, expensive tools, or long follow up. It would be implausible to conduct a trial in such a critical condition without reporting maternal death or morbidity. Our results indicate the need for adequately powered studies, where the sample size is calculated according to clinical outcomes. Large multi-center studies would be warranted to achieve the required power.

## Conclusions

In women with HELLP syndrome, the effect of corticosteroids versus placebo or no treatment is uncertain for critical patient-relevant outcomes. The currently available evidence does not support or refute the practice of corticosteroid administration for treating HELLP syndrome.

### Electronic supplementary material

Below is the link to the electronic supplementary material.

Supplementary Material 1

Supplementary Material 2

Supplementary Material 3

Supplementary Material 4

Supplementary Material 5

Supplementary Material 6

## Data Availability

All data relevant to this study are publicly available. Data, analysis script and materials related to this study are publicly available on the Open Science Framework at https://osf.io/9vwdq. The study protocol and materials were registered on September 18, 2023 at https://osf.io/yzku5. To facilitate reproducibility, this manuscript was written by interleaving regular prose and analysis code using R Markdown.
