# Supplementary material for: Corticosteroids for improving patient-relevant outcomes in HELLP syndrome: a systematic review and meta-analysis
Source: BMC Pregnancy Childbirth. 2024 Jul 18;24:487. doi: 10.1186/s12884-024-06665-y (PMC11264471; doi:10.1186/s12884-024-06665-y)
Supplement: Supplementary file 3 — Supplementary Material 3 [file 12884_2024_6665_MOESM3_ESM.pdf]

Corticosteroids for HELLP

Updated Search strategy
Date: February 3, 2024

PubMed

(("randomized controlled trial"[pt] OR "controlled clinical trial"[pt] OR randomized[tiab] OR placebo[tiab] OR "clinical trials as topic"[Mesh:NoExp] OR randomly[tiab] OR trial[ti]) NOT (animals[Mesh] NOT humans[Mesh])) AND ("HELLP Syndrome"[Mesh] OR "HELLP Syndrome"[tiab] OR (hemolysis[tiab] AND "low platelets"[tiab] AND "elevated liver enzymes"[tiab])) AND (Steroids[Mesh] OR Glucocorticoids[PA] OR glucocorticoids[tiab] OR steroids[tiab] OR Dexamethasone[tiab] OR Betamethasone[tiab] OR Prednisolone[tiab] OR corticosteroids[tiab])

CENTRAL
([mh "HELLP Syndrome"] OR "HELLP Syndrome":ti,ab,kw OR (hemolysis:ti,ab,kw AND "low platelets":ti,ab,kw AND "elevated liver enzymes":ti,ab,kw)) AND ([mh Steroids] OR [mh Glucocorticoids] OR glucocorticoids:ti,ab,kw OR steroids:ti,ab,kw OR dexamethasone:ti,ab,kw OR betamethasone:ti,ab,kw OR Prednisolone:ti,ab,kw OR corticosteroids:ti,ab,kw)

Web of Science
((DT="randomized controlled trial" OR DT="controlled clinical trial" OR (TI=randomized OR AB=randomized) OR (TI=placebo OR AB=placebo) OR MH="clinical trials as topic" OR (TI=randomly OR AB=randomly) OR TI=trial) NOT (MHX=animals NOT MHX=humans))AND (MHX="HELLP Syndrome" OR (TI="HELLP Syndrome" OR AB="HELLP Syndrome") OR ((TI=hemolysis OR AB=hemolysis) AND (TI="low platelets" OR AB="low platelets") AND (TI="elevated liver enzymes" OR AB="elevated liver enzymes"))) AND (MHX=Steroids OR MHX=Glucocorticoids OR (TI=Glucocorticoids OR AB=Glucocorticoids) OR (TI=steroids OR AB=steroids) OR (TI=DexamethasoneOR AB=Dexamethasone) OR (TI=Betamethasone OR AB=Betamethasone) OR (TI=Prednisolone OR AB=Prednisolone) OR (TI=corticosteroids OR AB=corticosteroids))

Scopus
((INDEXTERMS ("randomized controlled trial") OR INDEXTERMS ("controlled clinical trial") OR TITLE-ABS(randomized) OR TITLE-ABS(placebo) OR INDEXTERMS("clinical trials as topic") OR TITLE-ABS(randomly) OR TITLE(trial)) NOT (INDEXTERMS(animals) NOT INDEXTERMS(humans))) AND (INDEXTERMS("HELLP Syndrome") OR TITLE-ABS("HELLP Syndrome") OR (TITLE-ABS(hemolysis)AND TITLE-ABS("low platelets") AND TITLE-ABS("elevated liver enzymes"))) AND (INDEXTERMS(Steroids) OR ALL(Glucocorticoids)OR TITLE-ABS(Glucocorticoids) OR TITLE-ABS(steroids) OR TITLE-ABS(Dexamethasone) OR TITLE-ABS(Betamethasone) OR TITLE-ABS(Prednisolone) OR TITLE-ABS(corticosteroids))
