# Supplementary material for: Corticosteroids for improving patient-relevant outcomes in HELLP syndrome: a systematic review and meta-analysis
Source: BMC Pregnancy Childbirth. 2024 Jul 18;24:487. doi: 10.1186/s12884-024-06665-y (PMC11264471; doi:10.1186/s12884-024-06665-y)
Supplement: Supplementary file 5 — Supplementary Material 5 [file 12884_2024_6665_MOESM5_ESM.pdf]

Outcome definitions in studies included in meta-analyses (shaded cells)

	Maternal death (time frame)	Liver morbidity (hematoma, rupture, failure)	Acute pulmonary edema	Acute renal failure	PLT transfusion	Perinatal death
**Fonseca 2005 [17]**	Not specified.<br>The duration of hospitalization of the 4 maternal deaths was excluded for the calculation of mean and median but was included and treated as censored data in survival analysis.		Physical examination and chest radiography	Serum creatinine >1.5 mg/dL	When surgery was indicated, 8 units of platelets were transfused to women with platelet counts <50,000/ mm3	
**Fonseca 2019 [21]**	There were 2 maternal deaths, both in the placebo group during the **first 12 h of study entry**; one death was secondary to a cerebrovascular accident during C-section, and the other death occurred in a patient with liver failure and severe hemolysis at enrollment.		Physical examination and chest radiography	Serum creatinine >1.5 mg/dL	When C-section was indicated, 8 units of platelets were given to women with platelet counts < 50,000/ mm3 at time of incision. The same amount of platelets was also given if the platelet count was ≤20000; or there was presence of bleeding gums, hematuria or bleeding from venipuncture sites	
**Katz 2008 [26]**	The 4 cases of death referred to 1 patient with acute pulmonary embolism, 1 case of death following acute renal failure, 1 case of death following sepsis, and 1 case in which the patient was admitted to the hospital in a conscious state but rapidly went into a coma and died less than **24 hours following admission**.		Clinical findings of dyspnea, stertor, tachycardia, and cyanosis not caused by other clinical processes that could explain these findings.	Oliguria (<400 mL/day) associated with a sudden increase in plasma urea and creatinine.		

**Ozer 2009 [24]**	Not specified	hepatic subcapsular hematoma, hepatic rupture	cardiopulmonary complications (pulmonary edema)	Renal (acute renal failure, acute tubular necrosis, need for dialysis)		
**van Runnard 2006 [19]**	1 patient in Placebo group – **11 days after delivery** due to sepsis and multiorgan failure.	Liver hematoma, liver rupture				Antenatal death or neonatal death in first week of life
**Vigil-De Gracia 1997 [25]**	Not specified.<br>One death in the control group, this patient died from massive cerebral hemorrhage. Her death was related to seizure with a platelet count of 23 000 before convulsion.					
**Yalcin 1998 [20]**				Not specified		
**Magann 1994 [22]**						Neonatal death

Inclusion criterion according to GA or postpartum days

Study ID	Treatment commenced	GA or postpartum days	Days postpartum
**Bouchnak 2005 [27]**	Postpartum	Not specified	
**Fonseca 2005 [17]**	Antepartum and Postpartum	>20 weeks (mean GA: 33.6 weeks, range 20-41)	First 3 days in puerperium
**Fonseca 2019 [21]**	Antepartum and Postpartum	> 20 weeks (mean GA: 33.85 weeks, range 21–41)	First 3 days in puerperium
**Kadanali 1997 [28]**	Antepartum	27-37 weeks	
**Katz 2008 [26]**	Postpartum	Not specified	Not specified
**Magann 1994 AP [22]**	Antepartum	24-37 weeks	Not specified
**Magann 1994 PP [18]**	Postpartum	Not specified	Immediate postpartum
**Mould 2006 [29]**	Postpartum	Not specified	Not specified
**Ozer 2009 [24]**	Antepartum	>20 weeks<br>Mean GA: Betamethasone 32.4 ± 4.5 weeks, Control 33.1 ± 3.7 weeks	Not specified
**van Runnard 2006 [19]**	Antepartum	< 30 weeks	No new recruitments postpartum
**Vigil-De Gracia 1997 [25]**	Postpartum	Dexamethasone: 32.82 ± 3.42 weeks, Control: 34.41 ± 2.81 weeks	No new recruitments postpartum
**Yalcin 1998 [20]**	Postpartum	Mean GA at delivery: 35.1 ± 2.9 weeks (range 29-38)	No new recruitments postpartum
**Du Plessis 2010 [16]**	Postpartum	Not specified	No new recruitments postpartum
**Borekci 2008 [15]**	Postpartum	Dexamethasone: 31.2 ± 4.8 weeks, Betamethasone: 33.8 ± 4.7 weeks, Control: 32.4 ± 4.9 weeks	
**Caliskan 2010 [23]**	Antepartum and Postpartum	27-37 weeks<br>Betamethasone: 33 ± 2.5 weeks<br>Control: 33.8 ± 3 weeks	

List of excluded reports

Study ID	Reason for exclusion
Katz 2013 [a]<br>*published protocol*	Terminated; recruitment slower than anticipated [https://clinicaltrials.gov/study/NCT00711841?tab=history]
Pourrat 2016 [b]	Did not meet inclusion criteria for participants; enrolled women with thrombocytopenia, not HELLP syndrome
Sangolli 2020 [c]	Ongoing trial

a. Katz L, Amorim M, Souza JP, Haddad SM, Cecatti JG. COHELLP: collaborative randomized controlled trial on corticosteroids in HELLP syndrome. Reproductive health. 2013;10:28.

b. Pourrat O, Dorey M, Ragot S, de Hauteclocque A, Deruelle P, Dreyfus M, et al. High-Dose Methylprednisolone to Prevent Platelet Decline in Preeclampsia: A Randomized Controlled Trial. Obstetrics & Gynecology. 2016;128:153.

c. Accessible through the Clinical Trials Registry - India (CTRI) website: https://ctri.nic.in/Clinicaltrials, by searching using the CTRI number: CTRI/2020/12/029730, as the keyword.

Antenatal administration of corticosteroids for fetal lung maturation

Study ID	Treatment commenced	Study included participants who received steroids for fetal lung maturation
**Bouchnak 2005 [27]**	Postpartum	Not specified
**Fonseca 2005 [17]**	Antepartum and Postpartum	Yes
**Fonseca 2019 [21]**	Antepartum and Postpartum	Yes
**Kadanali 1997 [28]**	Antepartum	Not specified
**Katz 2008 [26]**	Postpartum	Yes
**Magann 1994 AP [22]**	Antepartum	Yes
**Magann 1994 PP [18]**	Postpartum	Not specified
**Mould 2006 [29]**	Postpartum	Yes
**Ozer 2009 [24]**	Antepartum	Yes
**van Runnard 2006 [19]**	Antepartum	Yes
**Vigil-De Gracia 1997 [25]**	Postpartum	Not specified
**Yalcin 1998 [20]**	Postpartum	Not specified
**Du Plessis 2010 [16]**	Postpartum	Not specified
**Borekci 2008 [15]**	Postpartum	Yes
**Caliskan 2010 [23]**	Antepartum and Postpartum	Excluded

Sample size calculation for included studies

Study ID	Sample size calculation was powered based on
**Bouchnak 2005 [27]**	Not specified
**Fonseca 2005 [17]**	Duration of hospitalization
**Fonseca 2019 [21]**	Conditional probability of discharge, and composite morbidity (acute renal failure, pulmonary edema, infections, eclampsia and death)
**Kadanali 1997 [28]**	Not specified
**Katz 2008 [26]**	Platelet count
**Magann 1994 AP [22]**	Not specified
**Magann 1994 PP [18]**	Not specified
**Mould 2006 [29]**	Not specified
**Ozer 2009 [24]**	Platelet count
**van Runnard 2006 [19]**	Entry-delivery period
**Vigil-De Gracia 1997 [25]**	Not specified
**Yalcin 1998 [20]**	Not specified
**Du Plessis 2010 [16]**	Not specified
**Borekci 2008 [15]**	Not specified
**Caliskan 2010 [23]**	Not specified
