# Supplementary material for: Corticosteroids for improving patient-relevant outcomes in HELLP syndrome: a systematic review and meta-analysis
Source: BMC Pregnancy Childbirth. 2024 Jul 18;24:487. doi: 10.1186/s12884-024-06665-y (PMC11264471; doi:10.1186/s12884-024-06665-y)
Supplement: Supplementary file 6 — Supplementary Material 6 [file 12884_2024_6665_MOESM6_ESM.pdf]

PRESS Guideline — Search Submission & Peer Review Assessment

SEARCH SUBMISSION:

Searcher: A Nabhan, A Fahmy, H Bakry
Email: anabhan@med.asu.edu.eg
Date submitted: 30-08-2023
Date requested by: 02-09-2023

Systematic Review Title:

Corticosteroids for improving patient-relevant outcomes in HELLP syndrome

This search strategy is ...

x	My PRIMARY (core) database strategy — First time submitting a strategy for search question and database
	My PRIMARY (core) strategy — Follow-up review NOT the first time submitting a strategy for search question and database. If this is a response to peer review, itemize the changes made to the review suggestions
	SECONDARY search strategy— First time submitting a strategy for search question and database
	SECONDARY search strategy — NOT the first time submitting a strategy for search question and database. If this is a response to peer review, itemize the changes made to the review suggestions

Database: MEDLINE
Database provider: National Library of Medicine.
Platform: PubMed

Research Question

In women with HELLP syndrome, does the administration of a corticosteroids, as compared to placebo or no intervention, improve patient-relevant outcomes?

PICO Format

P	women with HELLP syndrome, whether ante-partum or postpartum
I	Any corticosteroid
C	Placebo or no intervention
O	Maternal death
S	RCT

Was a search filter applied?

☒ Yes

Cochrane Highly Sensitive Search Strategy for identifying randomized trials in MEDLINE: sensitivity- and precision-maximizing version (2008 revision); PubMed format. Accessed from: https://handbook-5-1.cochrane.org/chapter_6/box_6_4_b_cochrane_hsss_2008_sensprec_pubmed.htm

The search strategy, exactly as run, including the number of hits per line. [mandatory]

Search number	Query	Results
1	randomized controlled trial [pt]	600,241
2	controlled clinical trial [pt]	690,744
3	randomized [tiab]	671,683
4	placebo [tiab]	247,951
5	clinical trials as topic [mesh:noexp]	201,193
6	randomly [tiab]	416,321
7	trial [ti]	291,593
8	#1 OR #2 OR #3 OR #4 OR #5 OR #6 OR #7	1,558,348
9	animals [mh] NOT humans [mh]	5,149,120
10	#8 NOT #9	1,435,387
11	HELLP Syndrome [mh]	1,989
12	HELLP Syndrome [tiab]	2,628
13	#11 OR #12	3,128
14	Steroids [mh]	923,297
15	Glucocorticoids [pa]	207,870
16	Dexamethasone [tiab]	65,719
17	Betamethasone [tiab]	5,669
18	Prednisolone [tiab]	31,607
19	#14 OR #15 OR #16 OR #17 OR #18	989,774
20	#10 AND #13 AND #19	25

PEER REVIEW ASSESSMENT: Date completed: 07-09-2023

Reviewer: Ahmed Samir [Information specialist, Publication support office] Email: pso@med.asu.edu.eg

1. TRANSLATION

A ---No revisions x
B --- Revision(s) suggested ☐
C --- Revision(s) required ☐

If "B" or "C," please provide an explanation or example

2. BOOLEAN AND PROXIMITY OPERATORS

A ---No revisions	x
B --- Revision(s) suggested	☐
C --- Revision(s) required	☐

If "B" or "C," please provide an explanation or example:

3. SUBJECT HEADINGS

A ---No revisions	☐
B --- Revision(s) suggested	x
C --- Revision(s) required	☐

If "B" or "C," please provide an explanation or example: **Use corticosteroids in MeSH**

4. TEXT WORD SEARCHING

A ---No revisions	☐
B --- Revision(s)suggested	x
C --- Revision(s) required	☐

If "B" or "C," please provide an explanation or example: **Use corticosteroids in TW**

5. SPELLING, SYNTAX, AND LINE NUMBERS

A ---No revisions	x
B --- Revision(s)suggested	☐
C --- Revision(s) required	☐

If "B" or "C," please provide an explanation or example:

6. LIMITS AND FILTERS

If "B" or "C," please provide an explanation or example:

A ---No revisions	x
B --- Revision(s) suggested	☐
C --- Revision(s) required	☐

7. OVERALL EVALUATION

A ---No revisions	☐
B --- Revision(s) suggested	x
C --- Revision(s) required	☐

Additional comments: None.
