# Supplementary material for: Corticosteroids for improving patient-relevant outcomes in HELLP syndrome: a systematic review and meta-analysis
Source: BMC Pregnancy Childbirth. 2024 Jul 18;24:487. doi: 10.1186/s12884-024-06665-y (PMC11264471; doi:10.1186/s12884-024-06665-y)

Corticosteroids in HELLP syndrome

Data and synthesis script

2024-02-03

Dataset

id	ee	ne	ec	nc	factor	corticosteroid	outcome	RoB	zero
Van Runnard 2006	1	15	1	16	antepartum	prednisolone	Abruptio placenta	low	F
Magann 1994	3	12	3	13	antepartum	dexa	Apgar score at 5 minutes < 7	some concerns	F
Van Runnard 2006	1	16	2	17	antepartum	prednisolone	Apgar score at 5 minutes < 7	low	F
Ozer 2009	23	30	26	30	antepartum	beta	Cesarean section	low	F
Van Runnard 2006	15	15	14	16	antepartum	prednisolone	Cesarean section	low	F
Fonseca 2019	6	35	8	38	mixed	dexa	Composite morbidity	low	F
Van Runnard 2006	1	15	4	16	antepartum	prednisolone	Composite morbidity	low	F
Ozer 2009	1	30	0	30	antepartum	beta	Dialysis	low	F
Fonseca 2005	8	66	10	66	mixed	dexa	Eclampsia	low	F
Fonseca 2019	2	40	1	41	mixed	dexa	Eclampsia	low	F
Magann 1994	1	12	0	13	antepartum	dexa	ICH	some concerns	F
Van Runnard 2006	4	16	2	17	antepartum	prednisolone	ICH	low	F
Fonseca 2005	3	66	1	66	mixed	dexa	Maternal death	low	F
Fonseca 2019	0	42	2	45	mixed	dexa	Maternal death	low	F
Katz 2008	2	56	2	49	postpartum	dexa	Maternal death	low	F
Ozer 2009	0	30	0	30	antepartum	beta	Maternal death	low	T
Van Runnard 2006	0	15	1	16	antepartum	prednisolone	Maternal death	low	F
Vigil-De Gracia 1997	0	17	1	17	postpartum	dexa	Maternal death	high	F
Ozer 2009	0	30	1	30	antepartum	beta	Maternal liver morbidity	low	F
Van Runnard 2006	0	15	3	16	antepartum	prednisolone	Maternal liver morbidity	low	F
Fonseca 2005	3	66	1	66	mixed	dexa	Maternal pulmonary edema	low	F
Fonseca 2019	0	41	1	43	mixed	dexa	Maternal pulmonary edema	low	F
Katz 2008	2	56	5	49	postpartum	dexa	Maternal pulmonary edema	low	F
Ozer 2009	1	30	1	30	antepartum	beta	Maternal pulmonary edema	low	F
Fonseca 2005	6	66	8	66	mixed	dexa	Maternal renal failure	low	F
Fonseca 2019	1	37	6	42	mixed	dexa	Maternal renal failure	low	F
Katz 2008	9	56	12	49	postpartum	dexa	Maternal renal failure	low	F
Ozer 2009	2	30	3	30	antepartum	beta	Maternal renal failure	low	F
Yalcin 1998	3	15	3	15	postpartum	dexa	Maternal renal failure	some concerns	F
Van Runnard 2006	0	16	2	17	antepartum	prednisolone	Necrotizing enterocolitis	low	F
Magann 1994	3	12	1	13	antepartum	dexa	Neonatal RDS	some concerns	F
Van Runnard 2006	6	16	8	17	antepartum	prednisolone	Neonatal RDS	low	F
Magann 1994	1	12	3	13	antepartum	dexa	Perinatal death	some concerns	F
Van Runnard 2006	3	16	4	17	antepartum	prednisolone	Perinatal death	low	F
Fonseca 2005	12	66	10	66	mixed	dexa	Platelet transfusion	low	F
Fonseca 2019	12	42	15	45	mixed	dexa	Platelet transfusion	low	F

Synthesis

Pre-specified outcomes

1. Maternal Death
2. Liver morbidity (hematoma, rupture, failure)
3. Acute pulmonary edema
4. Acute renal failure
5. Dialysis
6. Platelet transfusion
7. Perinatal death

Maternal Death

Pairwise meta-analysis

```
                         RR            95%-CI %W(common) %W(random)
Fonseca 2005         3.0000 [0.3202; 28.1042]       25.4       25.4
Fonseca 2019         0.2141 [0.0106;  4.3328]       14.1       14.1
Katz 2008            0.8750 [0.1280;  5.9809]       34.5       34.5
Ozer 2009                NA                          0.0        0.0
Van Runnard 2006     0.3548 [0.0156;  8.0730]       13.0       13.0
Vigil-De Gracia 1997 0.3333 [0.0146;  7.6344]       13.0       13.0

Number of studies: k = 5
Number of observations: o = 449 (o.e = 226, o.c = 223)
Number of events: e = 12

                         RR           95%-CI     z p-value
Common effect model  0.7700 [0.2492; 2.3799] -0.45  0.6499
Random effects model 0.7700 [0.2492; 2.3799] -0.45  0.6499

Quantifying heterogeneity:
 tau^2 = 0 [0.0000; 7.4236]; tau = 0 [0.0000; 2.7246]
 I^2 = 0.0% [0.0%; 79.2%]; H = 1.00 [1.00; 2.19]

Test of heterogeneity:
    Q d.f. p-value
 2.64    4  0.6192

Details on meta-analytical method:
- Inverse variance method
- Restricted maximum-likelihood estimator for tau^2
- Q-Profile method for confidence interval of tau^2 and tau
- Continuity correction of 0.5 in studies with zero cell frequencies
```

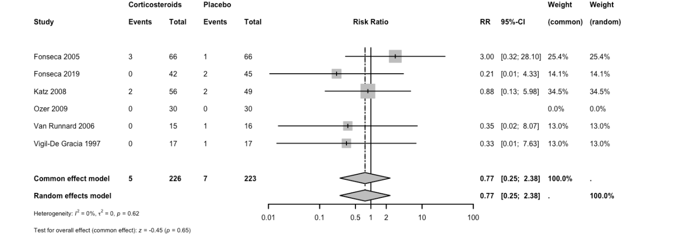

Risk of bias

Summary traffic-light plot of risk-of-bias assessments

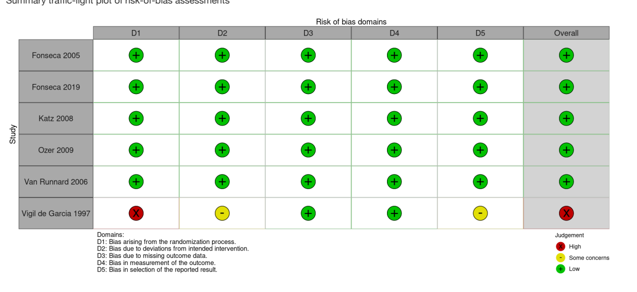

Summary weighted barplot of risk-of-bias assessments

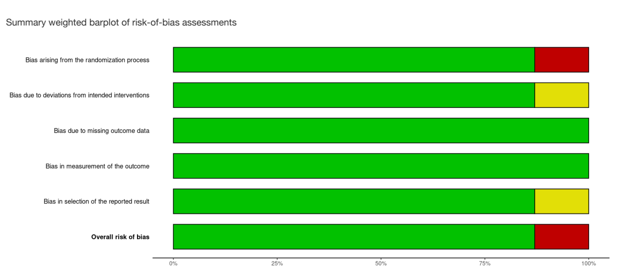

Subgroup analysis: Antepartum vs postpartum vs mixed

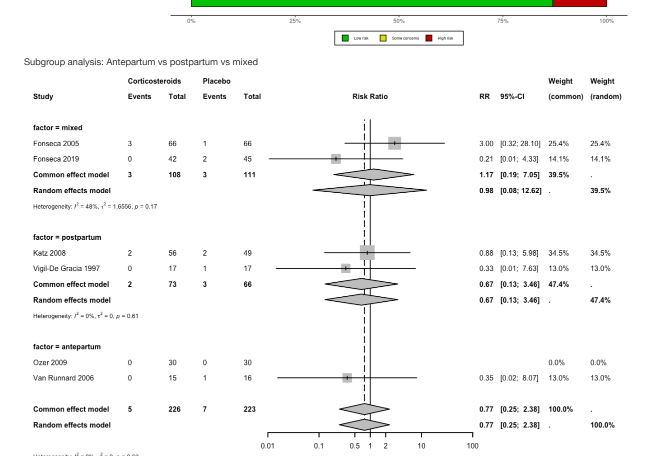

Subgroup analysis: Dexa vs Beta vs Prednisolone

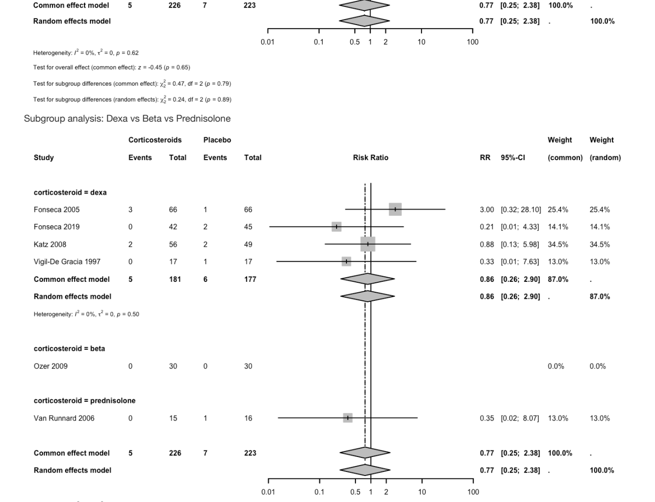

Sensitivity analysis: Low RoB

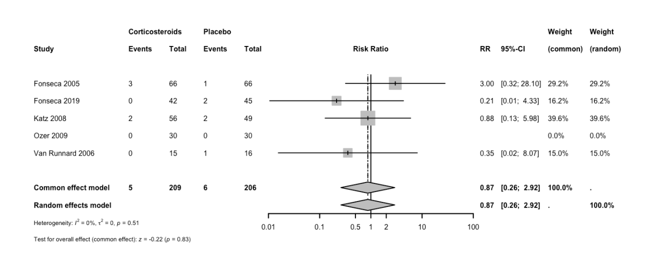

Sensitivity analysis: Studies with zero events included

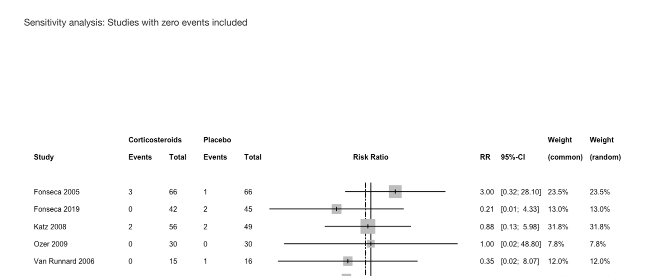

Funnel plot

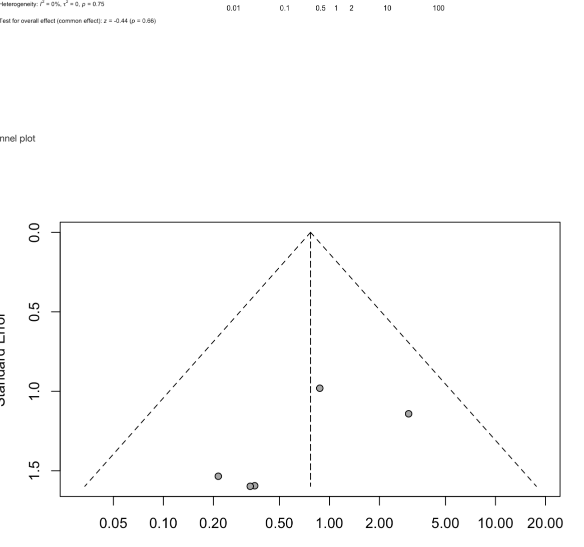

Acute pulmonary edema

```
                 RR            95%-CI %W(common) %W(random)
Fonseca 2005 3.0000 [0.3202; 28.1042]       24.2       24.8
Fonseca 2019 0.3494 [0.0146;  8.3368]       12.0       12.9
Katz 2008    0.3500 [0.0711;  1.7238]       47.6       45.2
Ozer 2009    1.0000 [0.0655; 15.2598]       16.3       17.2

Number of studies: k = 4
Number of observations: o = 381 (o.e = 193, o.c = 188)
Number of events: e = 14

                         RR           95%-CI     z p-value
Common effect model  0.6975 [0.2323; 2.0944] -0.64  0.5207
Random effects model 0.7136 [0.2231; 2.2817] -0.57  0.5693

Quantifying heterogeneity:
 tau^2 = 0.1164 [0.0000; 13.2337]; tau = 0.3412 [0.0000; 3.6378]
 I^2 = 0.0% [0.0%; 84.7%]; H = 1.00 [1.00; 2.56]

Test of heterogeneity:
    Q d.f. p-value
 2.60    3  0.4572

Details on meta-analytical method:
- Inverse variance method
- Restricted maximum-likelihood estimator for tau^2
- Q-Profile method for confidence interval of tau^2 and tau
- Continuity correction of 0.5 in studies with zero cell frequencies
```

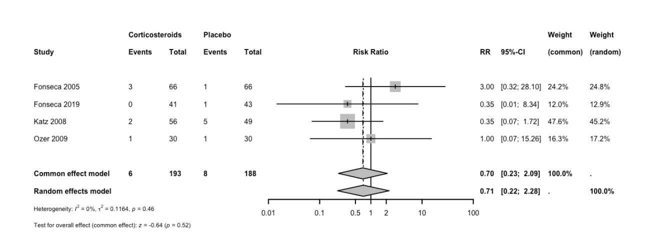

Acute renal failure

```
                 RR           95%-CI %W(common) %W(random)
Fonseca 2005 0.7500 [0.2754; 2.0428]       26.7       26.7
Fonseca 2019 0.1892 [0.0239; 1.4998]        6.3        6.3
Katz 2008    0.6563 [0.3025; 1.4238]       44.8       44.8
Ozer 2009    0.6667 [0.1198; 3.7087]        9.1        9.1
Yalcin 1998  1.0000 [0.2390; 4.1844]       13.1       13.1

Number of studies: k = 5
Number of observations: o = 406 (o.e = 204, o.c = 202)
Number of events: e = 53

                         RR           95%-CI     z p-value
Common effect model  0.6658 [0.3965; 1.1179] -1.54  0.1239
Random effects model 0.6658 [0.3965; 1.1179] -1.54  0.1239

Quantifying heterogeneity:
 tau^2 = 0 [0.0000; 2.4607]; tau = 0 [0.0000; 1.5687]
 I^2 = 0.0% [0.0%; 79.2%]; H = 1.00 [1.00; 2.19]

Test of heterogeneity:
    Q d.f. p-value
 1.78    4  0.7753

Details on meta-analytical method:
- Inverse variance method
- Restricted maximum-likelihood estimator for tau^2
- Q-Profile method for confidence interval of tau^2 and tau
```

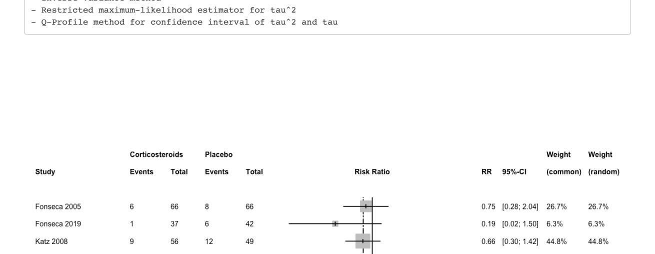

Dialysis

```
Number of observations: o = 60 (o.e = 30, o.c = 30)
Number of events: e = 1

              RR            95%-CI    z p-value
Ozer 2009 3.0000 [0.1271; 70.7833] 0.68  0.4958

Details:
- Continuity correction of 0.5
```


Liver morbidity

```
                     RR           95%-CI %W(common) %W(random)
Ozer 2009        0.3333 [0.0141; 7.8648]       45.4       45.4
Van Runnard 2006 0.1521 [0.0085; 2.7116]       54.6       54.6

Number of studies: k = 2
Number of observations: o = 91 (o.e = 45, o.c = 46)
Number of events: e = 4

                         RR           95%-CI     z p-value
Common effect model  0.2171 [0.0258; 1.8257] -1.41  0.1598
Random effects model 0.2171 [0.0258; 1.8257] -1.41  0.1598

Quantifying heterogeneity:
 tau^2 = 0; tau = 0; I^2 = 0.0%; H = 1.00

Test of heterogeneity:
    Q d.f. p-value
 0.13    1  0.7191

Details on meta-analytical method:
- Inverse variance method
- Restricted maximum-likelihood estimator for tau^2
- Continuity correction of 0.5 in studies with zero cell frequencies
```

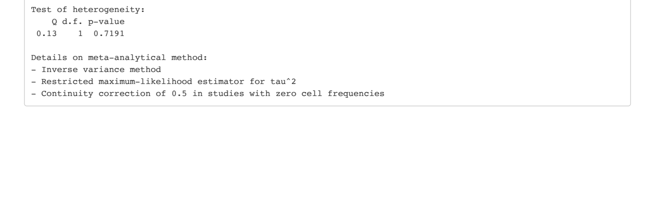

Platelet transfusion

```
                 RR           95%-CI %W(common) %W(random)
Fonseca 2005 1.2000 [0.5574; 2.5832]       40.5       40.5
Fonseca 2019 0.8571 [0.4556; 1.6126]       59.5       59.5

Number of studies: k = 2
Number of observations: o = 219 (o.e = 108, o.c = 111)
Number of events: e = 49

                         RR           95%-CI     z p-value
Common effect model  0.9821 [0.6031; 1.5994] -0.07  0.9422
Random effects model 0.9821 [0.6031; 1.5994] -0.07  0.9422

Quantifying heterogeneity:
 tau^2 = 0; tau = 0; I^2 = 0.0%; H = 1.00

Test of heterogeneity:
    Q d.f. p-value
 0.44    1  0.5069

Details on meta-analytical method:
- Inverse variance method
- Restricted maximum-likelihood estimator for tau^2
```

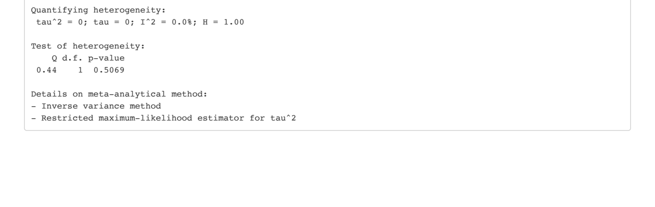

Perinatal death

```
                     RR           95%-CI %W(common) %W(random)
Magann 1994      0.3611 [0.0432; 3.0169]       28.3       28.3
Van Runnard 2006 0.7969 [0.2103; 3.0197]       71.7       71.7

Number of studies: k = 2
Number of observations: o = 58 (o.e = 28, o.c = 30)
Number of events: e = 11

                         RR           95%-CI     z p-value
Common effect model  0.6372 [0.2062; 1.9693] -0.78  0.4337
Random effects model 0.6372 [0.2062; 1.9693] -0.78  0.4337

Quantifying heterogeneity:
 tau^2 = 0; tau = 0; I^2 = 0.0%; H = 1.00

Test of heterogeneity:
    Q d.f. p-value
 0.38    1  0.5359

Details on meta-analytical method:
- Inverse variance method
- Restricted maximum-likelihood estimator for tau^2
```

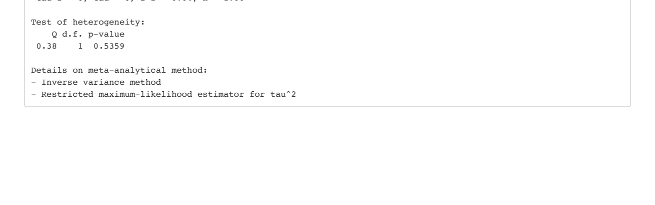

Supplement: Supplementary file 4 — Supplementary Material 4 [file 12884_2024_6665_MOESM4_ESM.pdf]
